# Tb-based silicate apatites showing slow magnetization relaxation with identical parameters for the Tb^3+^ and Dy^3+^ counter ions†

**DOI:** 10.1039/d1ra00613d

**Published:** 2021-02-12

**Authors:** Mikhail A. Zykin, Andrey K. Dyakonov, Artem A. Eliseev, Lev A. Trusov, Reinhard K. Kremer, Robert E. Dinnebier, Martin Jansen, Pavel E. Kazin

**Affiliations:** Department of Chemistry, Lomonosov Moscow State University 119991 Moscow Russia kazin@inorg.chem.msu.ru +7 495 9393440; Institute of General and Inorganic Chemistry RAS (IGIC RAS) 31 Leninsky Ave. 119991 Moscow Russia; Max Planck Institute for Solid State Research Heisenbergstrasse 1 70569 Stuttgart Germany

## Abstract

Tb-diluted and Tb-rich apatite-type silicates with compositions Y_7.75_Tb_0.25_Ca_2_(SiO_4_)_6_O_2_ and Tb_8_Ca_2_(SiO_4_)_6_O_2_, respectively, exhibit field induced multiple slow relaxation of magnetization. The former reveals two slow relaxation paths, the latter only one with a longer relaxation time of several seconds. The relaxation features of the Tb-diluted one are comparable with those of analogue compounds, where Tb is replaced by Dy, as well as with those of a Tb-doped calcium phosphate apatite. The relaxation parameters of the Tb-rich compound virtually match those of the Dy-based analogue Dy_8_Ca_2_(SiO_4_)_6_O_2_. The latter represents the first instance of independence of magnetization relaxation on the nature of a paramagnetic rare-earth metal ion in single ion magnet like materials.

## Abstract 2

Replacing Dy^3+^ with Tb^3+^ in the apatite-type silicate (Dy,Y)_8_Ca_2_(SiO_4_)_6_O_2_ preserves the character of the magnetization slow relaxation.

## Graphical abstract

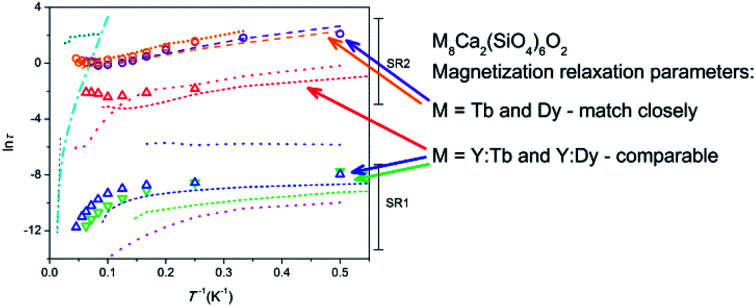

## Introduction

The research field of single molecule magnets (SMMs) and its subfield of single ion magnets (SIMs) have been developing fast since their discovery in 1993.^1^ In particular, substantial progress was achieved very recently when dysprosium(iii) bis-cyclopentadienyl complexes were shown to possess an energy barrier for magnetization reversal *U*_eff_ of above 1000 cm^−1^ and a magnetization blocking temperature *T*_b_ of 60 K,^2,3^ the latter value exceeded most recent previous values several times over. By now the current record for this kind of compounds is *U*_eff_ = 1541 cm^−1^ and *T*_b_ = 80 K.^4,5^ Such materials are considered as prospective candidates for molecular electronics, ultrahigh density data storage devices,^6,7^ spintronics,^8–11^ and quantum computers.^12,13^ The compounds of such functionality represent mostly complexes of open shell transition metals with organic ligands.^14–16^

As an alternative to the single-molecule metal-complex based materials, so called “all inorganic SIMs” have been developed.^17^ They represent lone ions of transition metals embedded in a diamagnetic and dielectric extended solid, which show a substantially superior chemical and thermal stability. A crystal of such a compound may serve as a substrate carrying a complete array of SIMs. A family of solids based on the hydroxyapatite Ca_10_(PO_4_)_6_(OH)_2_ structural matrix comprises the main representatives of such materials. In particular, slow relaxation of magnetization is observed for isolated dioxometalate-anions [OMO]^*n*−^ imbedded in the trigonal channels of alkaline earth phosphate apatites, where M = Cu,^18,19^ Ni,^20^ and Co.^21–23^ The dioxocobaltate(ii)-anion is found to perform the best. It shows slow relaxation under a zero magnetic field and *U*_eff_ reaches 387 cm^−1^ in a barium phosphate compound.^22^ The value is comparable with a record value of 450 cm^−1^ obtained recently for a linear complex of cobalt(ii).^24^ Another striking opportunity arises due to the fact that, in the apatite structure, not just the intrachannel atom site, but the M2 site as well has strongly anisotropic crystal environment. A lanthanide-ion replacing an alkaline earth metal ion at this site causes further distortion of the coordination sphere resulting in the formation of a LnO^+^ cation.^25^ The phosphate apatites containing a small fraction of “dysprosyl” DyO^+^ reveal SIM properties with *U*_eff_ reaching 1043 cm^−1^.^26^ This is not far from the current absolute record for SMMs, mentioned above. Tb^3+^ seems to be a second promising candidate to Dy^3+^ to form SIMs with high *U*_eff_ exemplified by its phthalocyanine complexes.^27,28^ However a similar TbO^+^ cation in the phosphate apatite matrix shows only a field-induced slow relaxation with a disappointingly low *U*_eff_.^29^

The phosphate apatite structure can accommodate only a very limited fraction of rare earth metal cations. In contrast to this, a silicate apatite may contain Ln^3+^ as a main constituent of cationic sublattice.^30^ This enables to realize a high concentration of anisotropic paramagnetic centres in the crystal, and to probe the effect of the inter-centres' interactions, which may be useful for developing quantum bits. In our recent papers we reported on synthesis and properties of Dy^3+^–Y^3+^ based silicate-apatites.^31,32^ They reveal multiple slow relaxation of magnetization induced by an external magnetic field. Compounds with Dy^3+^ as a main cation in the structure exhibit the longest relaxation time. Essential features of this relaxation process are a weak dependence of the relaxation time on field and temperature, its stabilization by a strong magnetic field, and apparent suppression of Raman and Orbach processes at elevated temperatures.^32^ In the present paper we report synthesis and magnetic properties of silicate-apatites containing Tb^3+^. This allows us to compare and find regularities in magnetization relaxation properties of a full set of compounds – apatite type phosphates and silicates with the Kramers Dy^3+^ and non-Kramers Tb^3+^ ions.

## Experimental

The compounds with the compositions Y_7.75_Tb_0.25_Ca_2_(SiO_4_)_6_O_2_ (1) and Tb_8_Ca_2_(SiO_4_)_6_O_2_ (2) were prepared in the form of sintered ceramics as follows. Analytical grade oxides, Tb_4_O_7_, Y_2_O_3_, SiO_2_, and calcium carbonate (Aldrich) were used as starting materials. Oxides were calcined in air at 1000 °C for 2 h to remove residual water and carbonate. Calcium carbonate was dried at 100 °C in air. The stoichiometric quantities of the starting materials were ground and mixed in an agate mortar. The mixtures were heat treated in air with the following schedule: heating to 400 °C within 1 h, keeping at this temperature for 1 h, heating to 600 °C within 1 h, keeping at this temperature for 1 h, heating to 800 °C within 1 h, and keeping at this temperature for 4 h. The procedure of the stepwise heating had to smooth possible primary reactions between the starting materials in order to prevent forming low-melting eutectics which may destroy homogeneity of the mixture. The products were ground and pressed in pellets with a pressure of *ca.* 5 × 10^3^ kg cm^−2^.

### Thermal treatment for 1

The pellets were heated to 1450 °C within 1 h, annealed at this temperature for 4 h, and air quenched. The obtained samples were reground, pressed in pellets, and heat treated at 1450 °C and then at 1580 °C in the same way with intermediate regrindings and pressings.

### Thermal treatment for 2

The pellets were heated to 1300 °C within 1 h, annealed at this temperature for 8 h, and air quenched. The obtained samples were reground, pressed in pellets, and heat treated at 1450 °C for one time and then at 1580 °C for two times in the same way with intermediate regrindings and pressings.

The samples represented dense and rather hard ceramics of white colour.

X-ray powder diffraction (XRPD) was carried out using D8 Brucker Advance powder diffractometers with Bragg–Brentano geometry using Cu-Kα1,2 (for 1) and Cu-Kα1 (for 2) radiations. 2*θ* was scanned in the range 10–100°. The crystal structures were refined using the Jana 2006 computer program.^33^ The powder profile, unit cell, and atomic (coordinates, atomic displacements) parameters were refined. The atomic displacement parameters we refined as anisotropic ones for M1 and M2 and isotropic ones for all other sites. The atomic ratios of metal cations were fixed to the nominal ones and occupancies of metal sites by Ca and Tb or Y–Tb were refined without vacancies at the sites and using the scattering cross-sections for charged species Tb^3+^, Y^3+^, Ca^2+^, O^−^, and neutral Si. In 1, Tb was assumed to follow Y with a fixed ratio corresponding to the nominal composition.

Magnetic measurements were performed on Quantum Design MPMS-XL-7 and PPMS-9 Magnetometers using a small ceramic piece of a sample firmly fixed in a sample holder. The field dependence of magnetization *M*(*H*) was measured at *T* = 2 K in the field range of 1–70 kOe at a field sweeping rate of 2 kOe min^−1^. The dc susceptibility *χ*(*T*) was measured in the temperature range of 2–300 K under magnetic fields of 1 and 10 kOe, and in the temperature range of 2–50 K under magnetic fields of 4.5, 5.5, 7, and 9 kOe. ac susceptibility as *χ*_ac_ = *χ′* − i*χ*′′ was measured at ac field amplitudes of 1–5 Oe in the frequency range of 0.1–1488 Hz (MPMS) for 2 and 10–10 000 Hz (PPMS) for 1 in the temperature range of 2–50 K under dc fields of 0, 1, 4, and 8 kOe. The magnetization and susceptibility values were corrected for core diamagnetism using Pascal's increments and for the contribution of the sample holder magnetization. A small non-linearity in *M*(*H*) developing at high temperatures was observed in 1, designating presence of a trace of a ferromagnetic impurity. The susceptibility values were corrected for this impurity assuming that its magnetization was independent on temperature and practically saturated under a field of 1 kOe. The modelling of crystal field parameters and magnetic properties were conducted using the PHI^34^ and CONCORD (a version of CONDON)^35,36^ computer programs. A primary crystal field analysis was performed applying the point-charge model.

The parameters of magnetization relaxation were determined by fitting simultaneously the frequency (*f*) dependence of real *χ*′ and imaginary *χ*′′ parts of ac susceptibility in the generalized Debye model.^37^ The estimated parameters were the contributions from slow relaxing centres (SR1, SR2) to a net susceptibility, the relaxation time *τ* and the relaxation time distribution width *α* for each kind of slow relaxing centres, and the adiabatic susceptibility which was considered as a contribution from fast relaxing centres (FR). In order to estimate *τ* of a low-frequency relaxation when maximum of *χ*′′ was below the lower frequency limit, additional data on a dc differential magnetization were used. The ac susceptibility value *χ*_0_ at a frequency approaching zero was fixed to the measured dc differential susceptibility. It allowed plausible estimation of *τ* and *α* with larger error bars though. A more detailed description on the magnetic data treatment and parameters evaluation can be found in ref. 32.

Scanning electron microscopy with energy dispersive X-ray spectroscopy analysis (EDS) was conducted using a Supra 50 VP LEO scanning electron microscope.

## Results and discussion

### Crystal structure

XRPD patterns of 1 and 2 are presented in Fig. S1 and S2.† The crystal structure details are collected in Tables S1–S6.† Both samples represent X-ray pure apatite-type phases. This limits the amounts of possible traces of crystalline phases to quantities corresponding to 0.2 mass%. EDS analysis supports the relative contents of calcium, rare earth elements, and silicon to correspond to the nominal ones within an accuracy of 5%. As at the preparation conditions any evaporation of the components is not to be expected, we regard the compositions of the compounds to be exactly equal to their nominal compositions of the respective starting mixtures. The apatite cell parameters are *a* = 9.3486(1) Å, *c* = 6.7883(1) Å, *V* = 513.79(1) Å^3^ for 1 and *a* = 9.3918(1) Å, *c* = 6.8552(1) Å, *V* = 523.65(1) Å^3^ for 2. The small increase of the parameters from 1 to 2 is connected to a slightly larger radius of Tb^3+^ in comparison with Y^3+^, 1.040 and 1.019 Å for c. n. 8, respectively.^38^ Earlier studied Dy_8_Ca_2_(SiO_4_)_6_O_2_ is characterized by *a* = 9.3654(2) Å, *c* = 6.8188(1) Å, *V* = 517.95(2) Å^3^,^31^ which is well in the middle reflecting that Dy^3+^ has an intermediate radius (1.027 Å).^38^

**Fig. 1:**
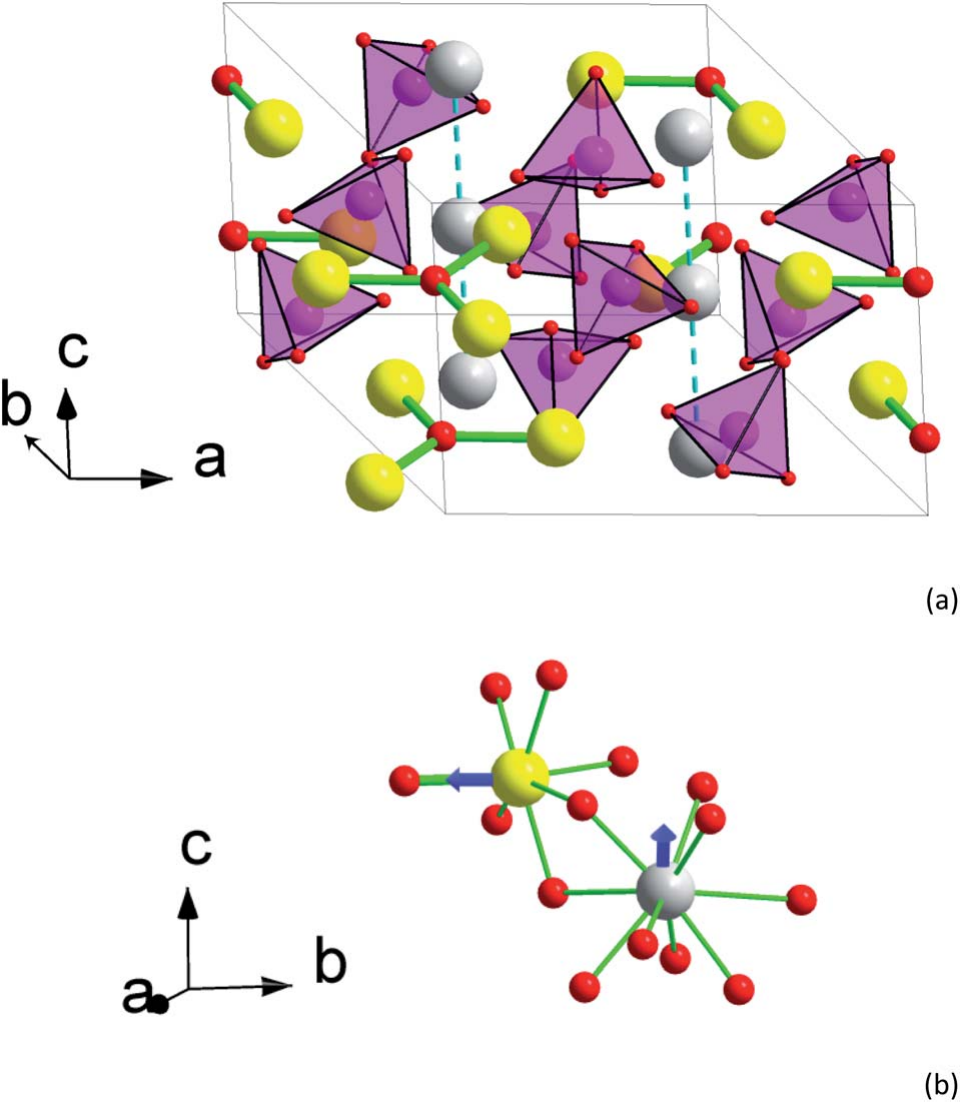
Crystal structure of 2. (a) Perspective view of the unit cell with an expansion to the trigonal channel (left front corner). (b) A fragment of the structure showing first coordination spheres of M1 and M2 atoms. Yellow – M2 atoms (Tb_0.944_Ca_0.056_), grey – M1 atoms (Tb_0.583_Ca_0.417_), magenta – Si, red – O, light magenta tetrahedrons – SiO_4_ groups. Tb–O bonds in Tb_3_O groups are shown as thick green sticks. Other Tb–O contacts are shown as thin green sticks in (b) only. Blue arrows designate directions of the magnetic moment in the ground pseudo-doublet of Tb^3+^.

The crystal structure of 2 is shown in Fig. 1. There are two sites for metal cations in the apatite structure, M1 (4f) and M2 (6h). The M2 atoms form “walls” of trigonal channels running along the *c* axis. Separate O^2−^ anions (O4) fill the center of the channel. The M2 site is practically fully occupied by the rare earth metal atoms, *ca.* 95% of Y,Tb and Tb in 1 and 2, respectively. The remaining 5% are for the Ca atoms. As a result, flat triangles Tb_3_O form in the *ab* plain with a Tb–O distance of 2.217 Å. Each Tb in Tb_3_O further coordinates to 6 oxygen atoms belonging to silicate groups, such that a distorted pentagonal bipyramid forms with O4 as one of the apexes. The distances to these oxygen atoms are longer and vary from 2.30 to 2.71 Å. As a result, the crystal field (CF) at M2 acquires a strong axial component determined mostly by the short M2–O4 bond.

The M1 site atoms form linear chains along the *c* axis. The site is filled by comparable quantities of calcium and rare earth metal atoms. The coordination sphere represents a set of oxygen atoms of silicate groups forming a twisted trigonal prism with its sides capped by three more distant oxygen atoms. The CF thus shows an axial anisotropy along *z*-direction.^31^

The interatomic contacts of Tb in the structure of 2 are slightly larger than those of Dy in Dy_8_Ca_2_(SiO_4_)_6_O_2_ [ref. 31] reflecting the cation size difference. Thus, the Dy–O distance in the Dy_3_O triangle is of 2.210 Å, being by 0.007 Å smaller than that in Tb_3_O. In 2 for Tb at M2, the average distance between Tb and silicate oxygen atoms is 2.405 Å in comparison with 2.389 Å in Dy_8_Ca_2_(SiO_4_)_6_O_2_. Similarly, an average distance between M1 and the coordinated oxygen atoms is 2.522 and 2.517 Å, respectively. This allows adequately comparing dc and ac magnetic properties of Tb and Dy at close CF parameters.

### Magnetization and dc susceptibility

**Fig. 2:**
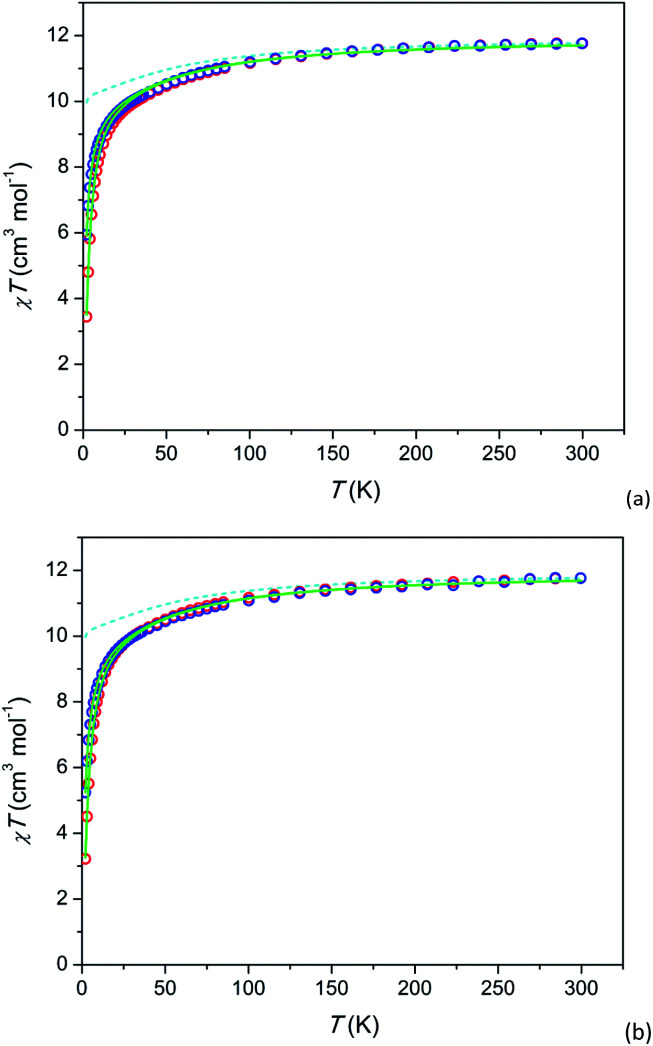
Temperature dependence of dc susceptibility-temperature product *χT* of 2 (a) and 1 (b) under fields of 1 and 10 kOe (blue and red circles respectively). Calculated *χT*(*T*): without inter-ion interaction (*H* = 1 kOe) – cyan dashed lines, with inter-ion interaction – full green lines. The data for 1 are scaled (multiplied) by a factor of 0.88 for better comparison.

**Fig. 3:**
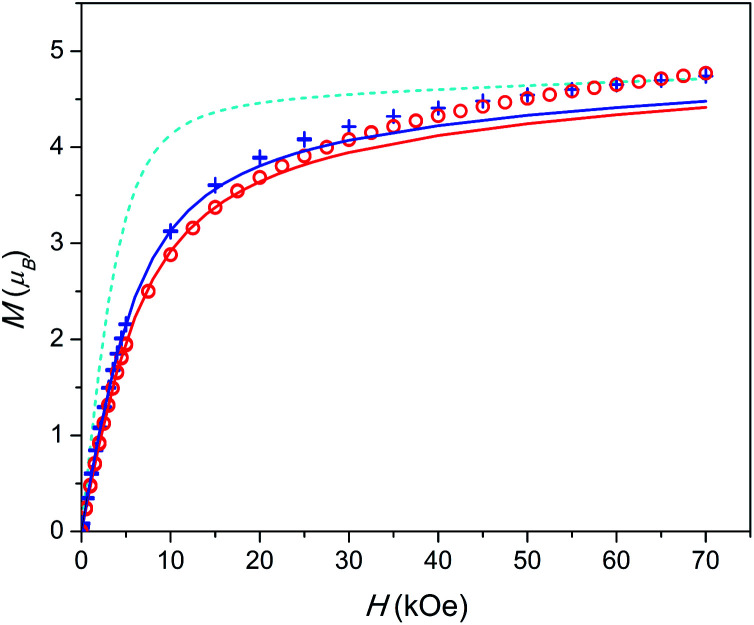
Field dependence of magnetization at *T* = 2 K. 1 – red circles, 2 – blue plus-signs. Calculated *M*(*H*): without inter-ion interaction – cyan dashed line, with inter-ion interaction – red (for 1) and blue (for 2) full lines. Data for 1 are scaled as in Fig. 2.

Temperature dependence of the susceptibility-temperature product *χT* under *H* = 1 and 10 kOe and field dependence of magnetization *M* at *T* = 2 K are shown in Fig. 2 and 3, respectively. The magnetization at 2 K is reversible implying that a possible relaxation time of magnetization cannot exceed a few tens of seconds. In 2 (Tb-rich) at a high temperature, *χT* approaches a value of 11.5 cm^3^ K mol^−1^ which is close to 11.8 cm^3^ K mol^−1^ for a free Tb^3+^ ion (^7^F_6_ ground state multiplet). With lowering temperature, the product decreases as expected due to depopulation of upper energy components of the ^7^F_6_ ground term split in the crystal field. In 1 (Tb-diluted) absolute values *χT* at high *T* slightly exceed expected ones. We connect it to lower measurement accuracy due to low content of Tb^3+^ in the compound and to somewhat inhomogeneity in the Tb^3+^ distribution over the sample.

For the further analysis we calculated the electronic structure of Tb^3+^ using the actual crystal structure data and taking partial charges at coordinated oxygen atoms as estimated for the Dy-based analogue compound^31^ (see Tables S7 and S8†). The theoretical *χT* values at *H* = 1 kOe are shown as dashed lines (cyan) in Fig. 2. The ground state is a pseudo-doublet with *M*_*J*_ = ±6 for Tb^3+^ at both, M1 and M2 sites. It corresponds to *χT* approaching 10 cm^3^ K mol^−1^ at low temperatures. However, the experimental values of low temperature *χT* are much smaller. *E.g.* for 2 at *T* = 2 K and *H* = 1 kOe, *χT* is about 6 cm^3^ K mol^−1^. A very similar behavior is observed in 1 (Tb-diluted). Under a field of 1 kOe χ*T* drops by more than a factor of 2 from *T* = 300 K to *T* = 2 K.

We assume that the considered strong drop of *χT* at low temperature may be accounted for by inter-ion dipolar or/and exchange interactions. Introducing of the effect of the interaction in the mean-field approximation (as implemented in PHI) results in good fits of all the *χT* curves (full green lines in Fig. 2). The mean-field parameters *zJ* are −0.047 cm^−1^ and −0.036 cm^−1^ for 1 and 2, respectively. This is surprising that Tb-rich and Tb-diluted compounds exhibit comparable values, the Tb-diluted compound displaying even larger |*zJ*|. One would expect that the dipolar interaction would be dozens times weaker in the Tb-diluted material. The close values of *zJ* may be connected to a certain compensation of inter-ion interactions in 2 as was discussed in ref. 31 for the Dy-rich sister compound, on one hand. On the other hand a relatively strong inter-ion interaction in 1 may be caused by a clustering of Tb^3+^ ions in M1 or/and M2 sites. Unfortunately the latter cannot be determined from the XRPD data since the Tb^3+^ ions are randomly distributed among the Y^3+^ ions. In contrast to 1 and 2 the analogue Dy-diluted and Dy-rich silicates exhibit only a moderate decrease of *χT* at low temperatures, so that their magnetic properties can be approximately considered as those of a magnetically isolated Dy^3+^ ion having the ground doublet with *M*_*J*_ = ±15/2.^31,32^

A similar situation is encountered when analysing the *M*(*H*) data (see Fig. 3). The theoretical *M*(*H*) curve without inter-ion interactions is far from the experimental data points. However, the curves, calculated using the *zJ* values determined from *χT*(*T*), follow well the experimental points at low magnetic fields. Their deviation at higher fields is quite expected since the mean-field approximation considers *χ* as a constant value not depending on *H*, and in this model, the magnetization under high fields cannot be properly analysed.

### Relaxation of magnetization

According to ac susceptibility data, the compounds do not show slow relaxation under a zero magnetic field even at a lowest temperature of 2 K. In 1 under a field of 1 kOe, a maximum on *χ*′′ and a respective drop of *χ*′ with increasing *f* arise (Fig. S3†). This corresponds to a part of magnetization relaxing slowly. It is denoted as a relaxation path SR1. In a higher field of 8 kOe the fraction of SR1 becomes larger (Fig. S4†). In addition to it, on the *χ*′(*f*) curve at a low frequency border an upward “tail” appears, which suggests the onset of relaxation with much longer *τ*. The comparison of the data with values of dc differential susceptibility (*χ*_0_) verifies that a considerable part of magnetization relaxes slower than SR1. This slower relaxation path is denoted as SR2. Two types of slow relaxation with similar properties have been registered in the analogue Dy-doped compound Y_7.75_Dy_0.25_Ca_2_(SiO_4_)_6_O_2_.^31^

In 2 under fields of 4 and 8 kOe, no relaxation in the intermediate frequency range is observed (Fig. S5 and S6†). However a similar low-frequency “tail” arises and the *χ*_0_ values become considerably larger than the *χ*′ values at the low frequency limit. This indicates that a very slow relaxation takes part for a fraction of magnetization. The relaxation behaviour looks very similar to that in the analogue Dy-based compound Dy_8_Ca_2_(SiO_4_)_6_O_2_,^31^ in which only one slow relaxation path SR2 is observed. The presence of one slow relaxation path in 2 and Dy_8_Ca_2_(SiO_4_)_6_O_2_ in comparison with two paths in the rare-earth metal diluted 1 and Y_7.75_Dy_0.25_Ca_2_(SiO_4_)_6_O_2_ may be related to a higher concentration of rare-earth metal ions and respectively to a lower inter-ion distance resulting in a considerable inter-ion dipolar interaction. As we discussed in [ref. 31], this interaction accelerates the magnetization relaxation of single-ion paramagnetic centers. Hence the expected second path (SR1) may not be detected, being out of the frequency range implied, thus contributing to an apparent fast relaxing part of magnetization. Whereas SR2 shows even enlarged relaxation time values in the Dy-rich compound, suggesting that a kind of inter-ion interactions, probably exchange interactions, stabilizes SR2. Such interactions (presumably of a ferromagnetic type) have been shown to cause considerable increase of magnetization relaxation time.^39–42^ A preliminary analysis of dipolar inter-ion interactions in Dy_8_Ca_2_(SiO_4_)_6_O_2_ resulted in a complex energy level system with inter-level spacing of an order of 1 cm^−1^.^32^ A possible presence of exchange coupling would entangle the situation even more. The details of interconnection between such interactions and the magnetization relaxation rate are not clear by now. For this reason we do not consider further effects of the inter-ion interactions on the relaxation of magnetization.

Thus, in 1, the net magnetization splits into three parts corresponding to SR1, SR2, and a fast relaxing path FR. Whereas in 2, it splits into two parts corresponding to SR2 and FR. The fractions of the magnetization involved in these slow relaxing paths increase with magnetic field and generally decrease with temperature so that at a certain temperature they drop to zero. In ref. 31 we discussed a possible origin of such multiple relaxation which is attributed to a single paramagnetic centre. In short, a strongly anisotropic paramagnetic centre may exhibit a specific dependence of relaxation time on magnetic field (a stepwise increase of *τ* with the field) and the field effect may be mostly determined by the field vector component *H*_*z*_ along the easy magnetization axis. As a result, for a sample representing an ensemble of randomly oriented crystallites, two or more peaks on *χ*′′(*f*) may appear characterizing different *τ* values for different intervals of *H*_*z*_.

**Fig. 4:**
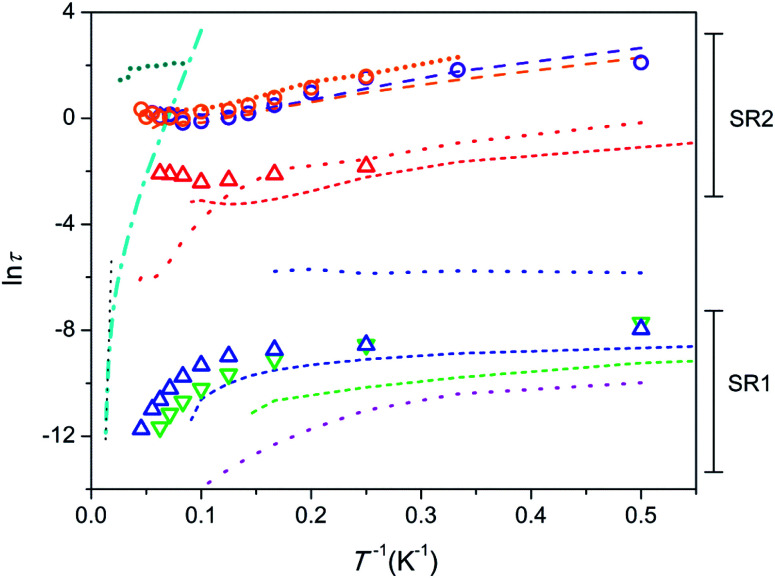
Dependence of ln *τ* (*τ* in seconds) on inverse temperature. 1 – triangles, 2 – circles; colour designation for 1: red – SR2 and *H* = 8 kOe, blue – SR1 and *H* = 8 kOe, green – SR1 and *H* = 1 kOe; colour designation for 2: orange – SR2 and *H* = 8 kOe, violet – SR2 and *H* = 4 kOe. Ca_9.5_Dy_0.5_(PO_4_)_6_(OH_0.75−*δ*_)_2_, *H* = 4 kOe^26^ – cyan dash-dot line; linear fit to show Orbach type relaxation – black dotted line. Ca_9.5_Tb_0.5_(PO_4_)_6_(OH_0.75−*δ*_)_2_ [ref. 29] – short-dash lines; colour designation: red – SR2 and *H* = 4 kOe, blue – SR1 and *H* = 4 kOe, green – SR1 and *H* = 1.5 kOe. Y_7.75_Dy_0.25_Ca_2_(SiO_4_)_6_O_2_ [ref. 31] – dotted lines; colour designation: red – SR2 and *H* = 4 kOe, blue – intermediate SR and *H* = 4 kOe, magenta – SR1 and *H* = 0. Dy_8_Ca_2_(SiO_4_)_6_O_2_, SR2 [ref. 31] – long-dash lines; colour designation: orange – *H* = 8 kOe, violet – *H* = 4 kOe. Dy_8_Mg_2_(SiO_4_)_6_O_2_, SR2 [32] – short-dot line; colour designation: dark-cyan – *H* = 50 kOe, orange – *H* = 8 kOe.

Temperature dependence of relaxation time in Arrhenius coordinates is shown in Fig. 4. The data points for 1 and 2 are depicted as symbols. In order to perform a comparative analysis, data points for similar apatite-type compounds containing Dy^3+^ and Tb^3+^, which we studied earlier,^26,29,31,32^ are presented also (as lines).

In fact, Tb^3+^ and Dy^3+^ happened to be best performing ions to form SIMs with a high energy barrier. Historically phthalocyanine complexes of Tb have been shown first to reveal promising SIMs properties.^27^ However later on, a large variety of Dy complexes including cyclopentadienyl complexes were prepared with substantially higher *U*_eff_ and *T*_b_.^2–5,28^ These ions may be considered as counter ions. Both ions display a similar oblate shape of f-electron shell, for which a linear two fold coordination provides a high easy-axis magnetic anisotropy. However Dy^3+^ is a Kramers ion having a half-integer spin of 5/2, while Tb^3+^ is non-Kramers ion with an integer spin of 3. As far as a Kramers doublet in the former cannot be split by CF, a low symmetry distortion of the coordination sphere does not affect much to aggravate the SIM properties. In contrary, such a distortion at Tb^3+^ splits a ground pseudo-doublet to release fast quantum tunnelling of magnetization. The latter strongly accelerates the relaxation especially at low temperatures.

Relaxation of magnetization in SMMs is considered to proceed *via* several different mechanisms.^43,44^ Quantum tunnelling of magnetization (QTM) and direct process determine *τ* at low temperatures, with the former acting stronger under low magnetic fields and the latter developing under higher fields. Raman process determines relaxation rate at intermediate temperatures, and thermally activated Orbach process prevails at higher temperatures providing an Arrhenius type dependence of *τ* on *T*. Temperature dependence of relaxation time can be expressed by eqn (1) which includes all listed processes as a series of respective terms.1
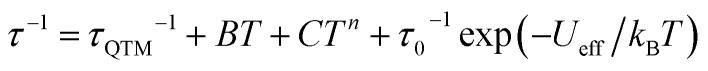


First we consider the Tb-diluted sample 1. *τ* values of SR1, both, under a field of 1 (green triangles in Fig. 4) and 8 kOe (blue triangles), being of a few parts of a ms at 2 K decrease with increasing *T* first slowly and then faster and faster so that eqn (1) may reflect this dependence. However, Orbach relaxation is not clearly indicated since ln *τ* (*T*^−1^) is far from following a straight line at the high temperature border. The dependence can be satisfactory fitted taking into consideration the QTM, direct, and Raman processes excluding a contribution from the Orbach type. The fit of SR1 under a field of 8 kOe yields τ_QTM_ = 6.4(17) × 10^−4^ s, *B* = 7.3(12) × 10^3^ s^−1^ K^−1^, *C* = 0.08(9) s^−1^ K^−*n*^, *n* = 4.6(4). The values obtained still show large uncertainties due to strong correlations between the varied parameters. Hence we will consider SR1 only qualitatively. The increase of the field hardly affects *τ* at low temperatures, but enhances *τ* at high temperatures. This may signify that the magnetic field suppresses the Raman process. *τ* of SR2 in 1 (red triangles) only weakly depends on temperature suggesting QTM to dominate.

Similar SR1 and SR2 paths have been found in Ca_9.5_Tb_0.5_(PO_4_)_6_(OH_0.75−*δ*_)_2_.^29^ For SR1, a similar enhancement of *τ* with increasing field, most pronounced at high temperatures, has been observed (green and blue short-dash lines in Fig. 4). And the SR2 paths in both, Ca_9.5_Tb_0.5_(PO_4_)_6_(OH_0_._75−*δ*_)_2_ (red short-dash line) and 1 exhibit comparable *τ* values.

Another material suited for comparison is the sister compound Dy-diluted Y_7.75_Dy_0.25_Ca_2_(SiO_4_)_6_O_2_ which shows three slow relaxation paths.^31^ The slowest one (red dotted curve) is stabilized by magnetic field and exhibit *τ* values of a fraction of a second at low temperatures. Therefore it is comparable with SR2 in 1, differing from the latter at temperatures above 10 K because of the fast drop of *τ* with increasing *T*. The intermediate slow one (blue dotted curve) seems to have properties different from SR1 and SR2 in 1. Its fraction drops fast with increasing magnetic field and temperature. The fastest one (magenta dotted curve) can be related to SR1 in 1, showing a similar decrease of *τ* with increasing temperature. It is characterized by at least one order of magnitude smaller values of *τ* though.

The Tb-rich sample 2 exhibits only SR2 which is characterized by *τ* values of several seconds at low temperature (orange and violet circles). With increasing temperature, *τ* first decreases by about one order of magnitude and then stabilizes at about 1 s. The temperature dependence of *τ* cannot be fitted by eqn (1). The *τ* values remain practically equal under different magnetic fields applied. A spectacular fact is that the *τ* values nearly coincide with those in the analogue Dy-based compound Dy_8_Ca_2_(SiO_4_)_6_O_2_ in the whole temperature and field ranges (orange and violet dashed lines).^31^ This suggests that the nature of the paramagnetic rare-earth metal cation does not play a role and the magnetization relaxation behavior is solely defined by the crystal structure which is very close for these two compounds. The other Dy-rich silicate apatite in which Mg replaces Ca shows similar features of SR2 with comparable *τ* (orange shot-dot line) and additionally, under a higher field of 50 kOe, larger *τ* values are found, which persist without appreciable variation up to 40 K (dark-cyan shot-dot line).^32^ These similarities imply that the diamagnetic cation only slightly affects the magnetization relaxation parameters.

As a next step it seems useful to compare all the compounds considered above against the background of the father compound Ca_9.5_Dy_0.5_(PO_4_)_6_(OH_0.75−*δ*_)_2_ which exhibits very different magnetization relaxation properties (Fig. 4, cyan dash-dot line). It shows a large energy barrier for remagnetization of 792 cm^−1^, a long relaxation time of hours under a magnetic field at 2 K, and *T*_b_ = 4.5 K.^26^ The SIM features are connected to the presence of the DyO^+^ cation with a short Dy–O bond of 2.14 Å, which provides a strong axial CF. Longer contacts to further six oxygen atoms of phosphate groups disturb slightly a perfect cylindrical axiality. Replacement of Dy by Tb in Ca_9.5_Tb_0.5_(PO_4_)_6_(OH_0_._75−*δ*_)_2_ results in a sharp suppression of the SIM behaviour.^29^ The interatomic distance in TbO^+^ is still low, of 2.15 Å, and the axial CF causes strong magnetic anisotropy favourable for the appearance of magnetic bistability. The poor SIM properties are most probably connected to the non-Kramers nature of Tb^3+^ so that low-symmetry components of CF split the ground pseudo-doublet opening QTM paths. Alternatively, inserting Dy in silicate apatites with the compositions Y_7.75_Dy_0.25_Ca_2_(SiO_4_)_6_O_2_ and Dy_8_Ca_2_(SiO_4_)_6_O_2_ results again in the suppression of SIM properties. This time Dy^3+^ shares neighbouring O^2−^ with Y^3+^ (Dy^3+^) instead of Ca^2+^. This causes the elongation of the Dy–O distance to 2.21 Å, weakening of the axiality, so that low symmetry components of CF become more pronounced. This may be sufficient to considerably enhance the magnetization relaxation rate.

One can see that the ln *τ*(*T*^−1^) plots for most of the samples considered lay well below the one for Ca_9.5_Dy_0.5_(PO_4_)_6_(OH_0.75−*δ*_)_2_ denoting their poor SIM properties. However ln *τ*(*T*^−1^) for the compounds with high concentration of Dy or Tb, namely Dy_8_Ca_2_(SiO_4_)_6_O_2_, Dy_8_Mg_2_(SiO_4_)_6_O_2_, and Tb_8_Ca_2_(SiO_4_)6O_2_, cross the ln *τ*(*T*^−1^) line for this high energy barrier SIM. This may indicate that Raman process prevailing at these temperatures is suppressed, while Orbach process is not activated yet. In this situation using a *τ* value measured at the highest temperature one can estimate a lower limit of a possible energy barrier for magnetization reversal. For 2, taking *τ*_0_ = 10^−12^ s (estimated for Ca_9.5_Dy_0.5_(PO_4_)_6_(OH_0.75−*δ*_)_2_)^26^ and a *τ* value under a field of 8 kOe, we get *U*_eff_ > 420 cm^−1^. The splitting of ^7^F_6_ of Tb^3+^ by CF is as follows (see Table S8†). The first exited pseudo-doublet having *M*_*J*_ = ±5 is at 141 and 131 cm^−1^ and the highest level (*M*_*J*_ = 0) of ^7^F_6_ is at 421 and 577 cm^−1^ for the M1 and M2 sites, respectively. Therefore the Orbach relaxation cannot process through the first and several next low lying exited states, although the participation of the upper states of ^7^F_6_ is still possible. A very similar situation was encountered for the Dy-based analogue Dy_8_Ca_2_(SiO_4_)_6_O_2_.^31^ For Dy_8_Mg_2_(SiO_4_)_6_O_2_ studied under much higher field of 50 kOe, the same procedure yielded *U*_eff_ > 872 cm^−1^.^32^ The latter value considerably exceeded a total energy splitting of the ground term of Dy^3+^ caused by CF (517 cm^−1^), suggesting that the Orbach process is partially forbidden. The *U*_eff_ estimates shown above are quite comparable with the energy barrier for remagnetization observed in Ca_9.5_Dy_0.5_(PO_4_)_6_(OH_0.75−*δ*_).

**Fig. 5:**
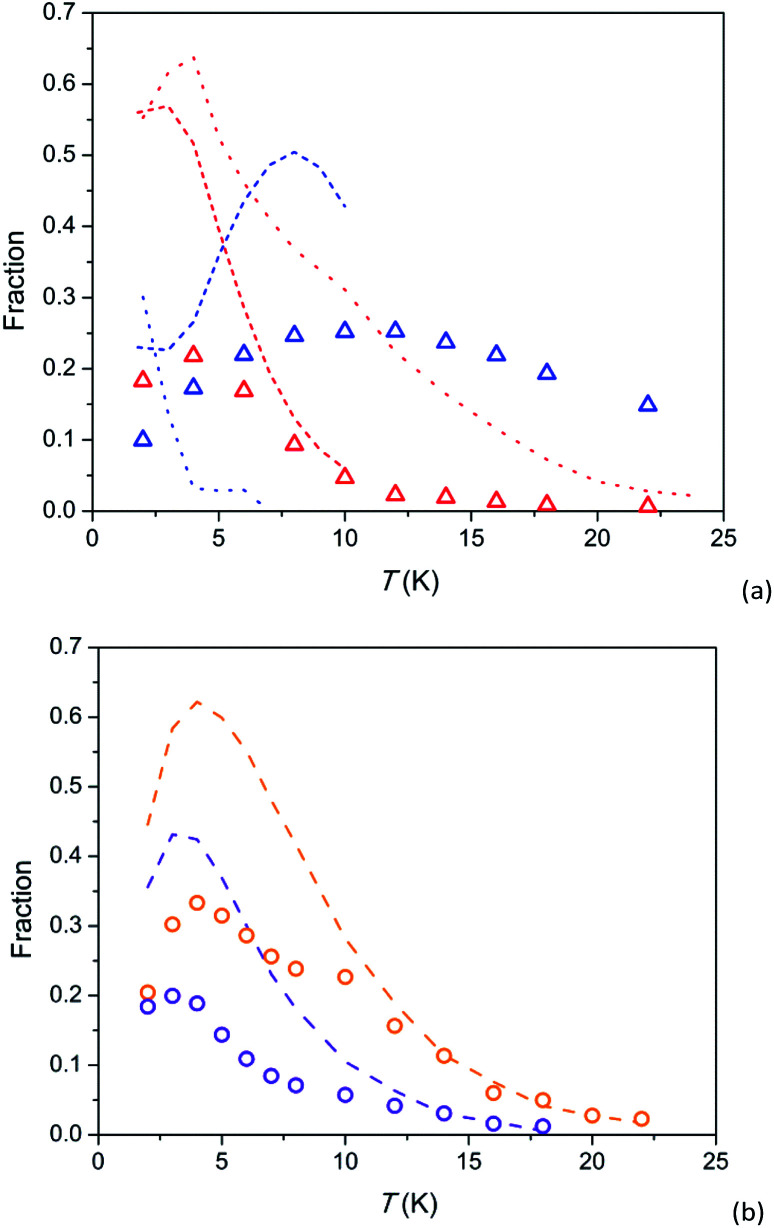
Temperature dependence of susceptibility fractions *F* for relaxation paths SR1 and SR2 in 1 (a) and 2 (b) and related compounds. 1 – triangles, 2 – circles; colour designation for 1: red – SR2 and *H* = 8 kOe, blue – SR1 and *H* = 8 kOe; colour designation for 2: orange – SR2 and *H* = 8 kOe, violet – SR2 and *H* = 4 kOe. Ca_9.5_Tb_0.5_(PO_4_)_6_(OH_0.75−*δ*_)_2_ [ref. 29] (a) – short-dash lines; colour designation: red – SR2 and *H* = 4 kOe, blue – SR1 and *H* = 4 kOe, green – SR1 and *H* = 1.5 kOe. Y_7.75_Dy_0.25_Ca_2_(SiO_4_)_6_O_2_ [ref. 31] (a) – dotted lines; colour designation: red – SR2 and *H* = 4 kOe, blue – intermediate SR and *H* = 4 kOe. Dy_8_Ca_2_(SiO_4_)_6_O_2_, SR2 [ref. 31] (b) – dashed lines; colour designation: orange – *H* = 8 kOe, violet – *H* = 4 kOe.

Further insight can be obtained by analysing magnetization fractions *F* involved in SR1 and SR2 processes in dependence on temperature (Fig. 5). Under a field of 8 kOe, SR1 and SR2 in 1 are simultaneously observed (blue and red triangles in Fig. 5a, respectively). *F*(SR2) after a small maximum at 4 K decreases fast with increasing temperature so that its contribution becomes hardly visible above 20 K. *F*(SR1) first grows with temperature up to 10–12 K and then decreases slowly retaining more than a half of its maximum value to 22 K. The behaviour is similar to that observed earlier in Ca_9.5_Tb_0.5_(PO_4_)_6_(OH_0.75−*δ*_)_2_ (short-dash lines), however SR1 and SR2 in 1 exhibit smaller *F* values and are observable to higher temperatures. Temperature dependence of *F*(SR2) in 1 is also resembles that in analogue Dy-based Y_7.75_Dy_0.25_Ca_2_(SiO_4_)_6_O_2_ (red dotted line), differing from it by smaller *F* values and their faster decrease with increasing temperature. A fraction of the intermediate relaxation path in the Dy-based analogue (blue dotted line) drops very fast with increasing temperature. Thus this path is clearly different from SR1 in 1, as we have mentioned above discussing temperature dependence of *τ*.

*F*(SR2) in 2 as a function of temperature has a similar shape as in 1: an increase to a maximum at 3–4 K and asymptotical-like decrease at higher temperatures (symbols in Fig. 5b). It is essential that the curves for 2 virtually coincide with the corresponding curves for the analogue Dy-based Dy_8_Ca_2_(SiO_4_)_6_O_2_ (dashed lines) if we multiply values of *F* by a factor of about 2. It is an additional evidence of very similar parameters of magnetization relaxation for the Tb and Dy analogues with an identical crystal structure. The fraction of magnetization involved in SR2 has been shown to increase with magnetic field up to 50 kOe.^32^ Hence it is not excluded that lower *F* for the Tb-based compound are connected to a stronger magnetic field required to stabilise the SR2 path. And a possible reason for the latter might be a lower magnetic moment of the ground pseudo-doublet.

Therefore the magnetization relaxation in the Tb-diluted silicate apatite 1 exhibits strong similarities with both, the Tb-diluted phosphate apatite and the Dy-diluted silicate apatite. Apparently the relaxation parameters are constrained by similar mechanisms in all these compounds. And the nature of the rare-earth paramagnetic ion has only a secondary value. Considering Tb-rich 2 and its analogue Dy-based compounds we may conclude that the magnetization relaxation features are almost totally specified by their crystal structure but the nature of the rare-earth metal cation. This peculiar relaxation phenomenon may arise due to a certain kind of inter-ion interaction in such compounds determined by their crystal structure.

## Conclusions

Single phase samples of silicates Y_7.75_Tb_0.25_Ca_2_(SiO_4_)_6_O_2_ and Tb_8_Ca_2_(SiO_4_)_6_O_2_ with apatite type structure were prepared by high temperature solid state method. Their crystal structures were refined using X-ray powder diffraction data. The crystal structure parameters are very close to those for the analogue Dy-based silicate apatites. Tb and Y are found to occupy practically fully the M2 site and share the M1 site with Ca ions. Modeling of electronic structure shows that Tb^3+^ in both M1 and M2 sites has a pseudo-doublet ground state with maximal |*M*_*J*_| = 6. The temperature dependence of susceptibility and field dependence of magnetization can be fitted using the modeled parameters and taking into account inter-ion interactions. The compounds reveal slow relaxation of magnetization under magnetic fields. Tb-diluted 1 exhibits multiple relaxation behavior with two slow relaxation paths, SR1 and SR2. Their parameters – temperature and field dependence of relaxation time and magnetization fractions involved in the relaxation processes, are comparable with those in the analogue Dy-based silicate and the Tb-doped phosphate. This finding suggests the relaxation mechanisms to be identical. Tb-rich 2 shows slow relaxation with the features of the SR2 path. All measured relaxation time values at different temperatures and fields are almost coincide with those observed in the analogue Dy-based silicate. Moreover, the fraction of slow relaxing magnetization follows the value in the Dy-based compound at different temperatures and fields, being about a factor of 2 lower. These facts imply that the magnetization relaxation in the Tb- and Dy-rich compounds does not depend on the nature of the paramagnetic rare-earth metal ion. The respective kind of relaxation possesses peculiar features: (i) the relaxation time of several seconds weakly dependent on field and temperature and (ii) the relaxation path is stabilized by magnetic field. Probably, inter-ion interactions being pronounced due to close distance between paramagnetic ions play an important role in controlling this type of relaxation. This kind of material may be of interest to build correlated quantum bits for quantum computers.

## Conflicts of interest

There are no conflicts to declare.

## Supplementary Material

RA-011-D1RA00613D-s001

RA-011-D1RA00613D-s002
