# Supplementary material for: Tb-based silicate apatites showing slow magnetization relaxation with identical parameters for the Tb3+ and Dy3+ counter ions
Source: RSC Adv. 2021 Feb 12;11(12):6926–33. doi: 10.1039/d1ra00613d (PMC8694933; doi:10.1039/d1ra00613d)
Supplement: RA-011-D1RA00613D-s001 [file RA-011-D1RA00613D-s001.pdf]

Electronic Supplementary Material (ESI) for RSC Advances.
This journal is © The Royal Society of Chemistry 2021

Electronic supplementary information

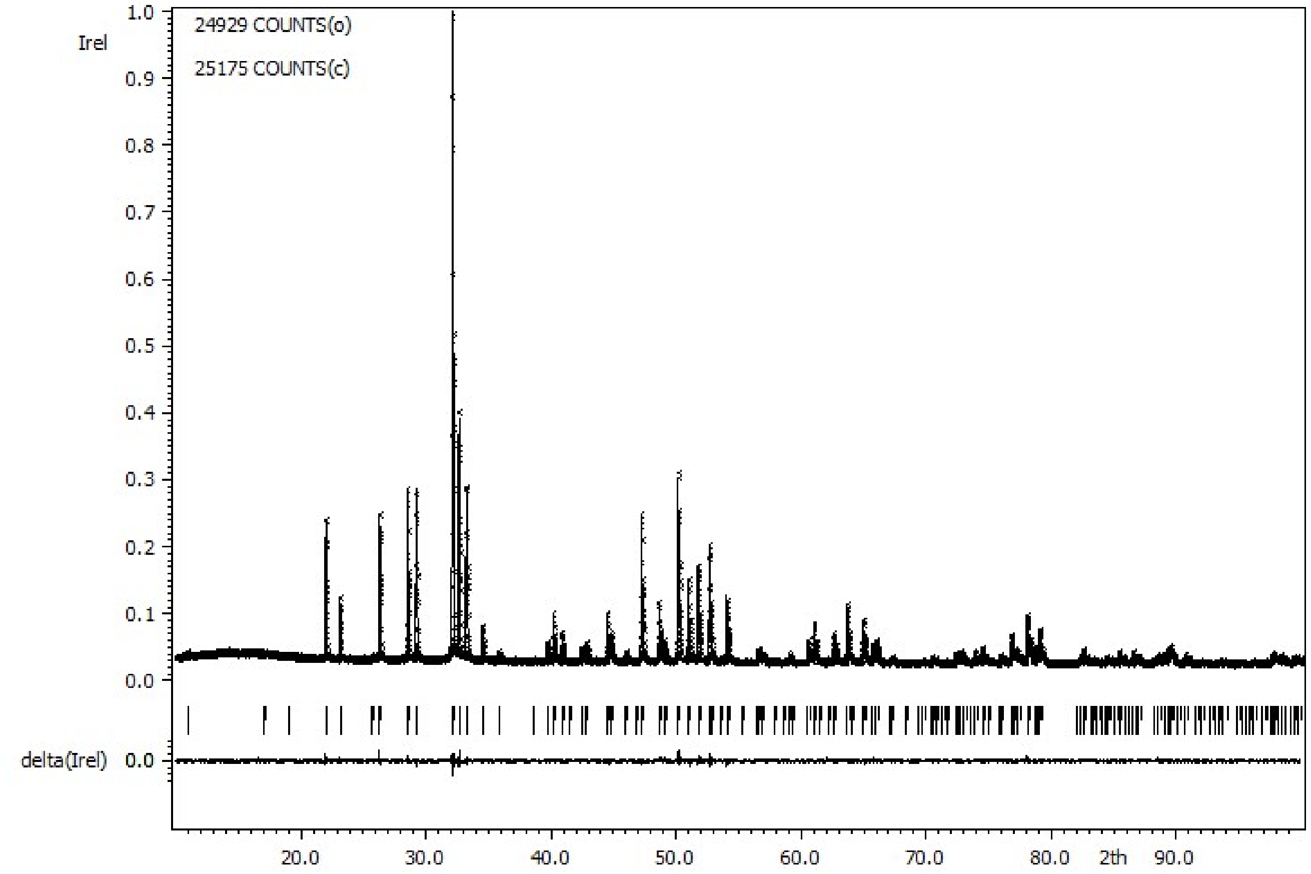

Figure S1. X-ray powder diffraction pattern of $Y_{7.75}Tb_{0.25}Ca_2(SiO_4)_6O_2$ (**1**). Observed (crosses), calculated (solid line) and difference (solid line below) plots. Positions of Bragg reflections are shown as strokes underneath.

Table S1. Crystal structure refinement data for **1**.

Temperature (K)	293 K
Wavelength (Å)	1.5418
Space group	$P6_3/m$
a (Å)	9.3486(1)
c (Å)	6.7883(1)
V (Å^3)	513.79(1)
Z	1
2θ range (deg.)	10 – 100
R_{wp}	0.036
R_{all}	0.024
ΔF_{max}, ΔF_{min} (e Å^{-3})	0.32, -0.45

Table S2. Atomic parameters and thermal displacement parameters (Å^2) in **1**.

Atom	Y1[a], Ca1	Y2[a], Ca2	Si	O1	O2	O3	O4
Site	4f	6h	6h	6h	6h	12i	2b
SOF	0.568(5), 0.432(5)	0.954(3), 0.046(3)	1	1	1	1	1
X	1/3	0.0040(2)	0.3994(4)	0.3178(8)	0.6006(8)	0.3370(6)	0
Y	2/3	0.23732(15)	0.3719(5)	0.4867(8)	0.4777(8)	0.2478(6)	0
Z	0.0013(5)	1/4	1/4	1/4	1/4	0.0612(5)	0.25
U_{iso}	0.0086(11)	0.0051(11)	0.0077(14)	0.0062(15)	0.0062(15)	0.0062(15)	0.0062(15)

[a] Y1, Y2 was refined as the mixture $Y_{0.96875}Tb_{0.03125}$.

Table S3. Selected interatomic distances (Å) and angles (degrees) in **1**.

Y1,Ca1-O1	2.336(6)	3x	Si-O1	1.598(11)	
Y1,Ca1-O2	2.439(7)	3x	Si-O2	1.629(8)	
Y1,Ca1-O3	2.802(5)	3x	Si-O3	1.628(5)	2x
Y2,Ca2-O1	2.684(6)		O1-Si-O2	112.7(4)	
Y2,Ca2-O2	2.345(12)		O1-Si-O3	110.9(3)	2x
Y2,Ca2-O3	2.286(4)	2x	O2-Si-O3	109.0(3)	2x
Y2,Ca2-O3	2.417(5)	2x	O3-Si-O3	103.8(3)	
Y2,Ca2-O4	2.2004(17)				

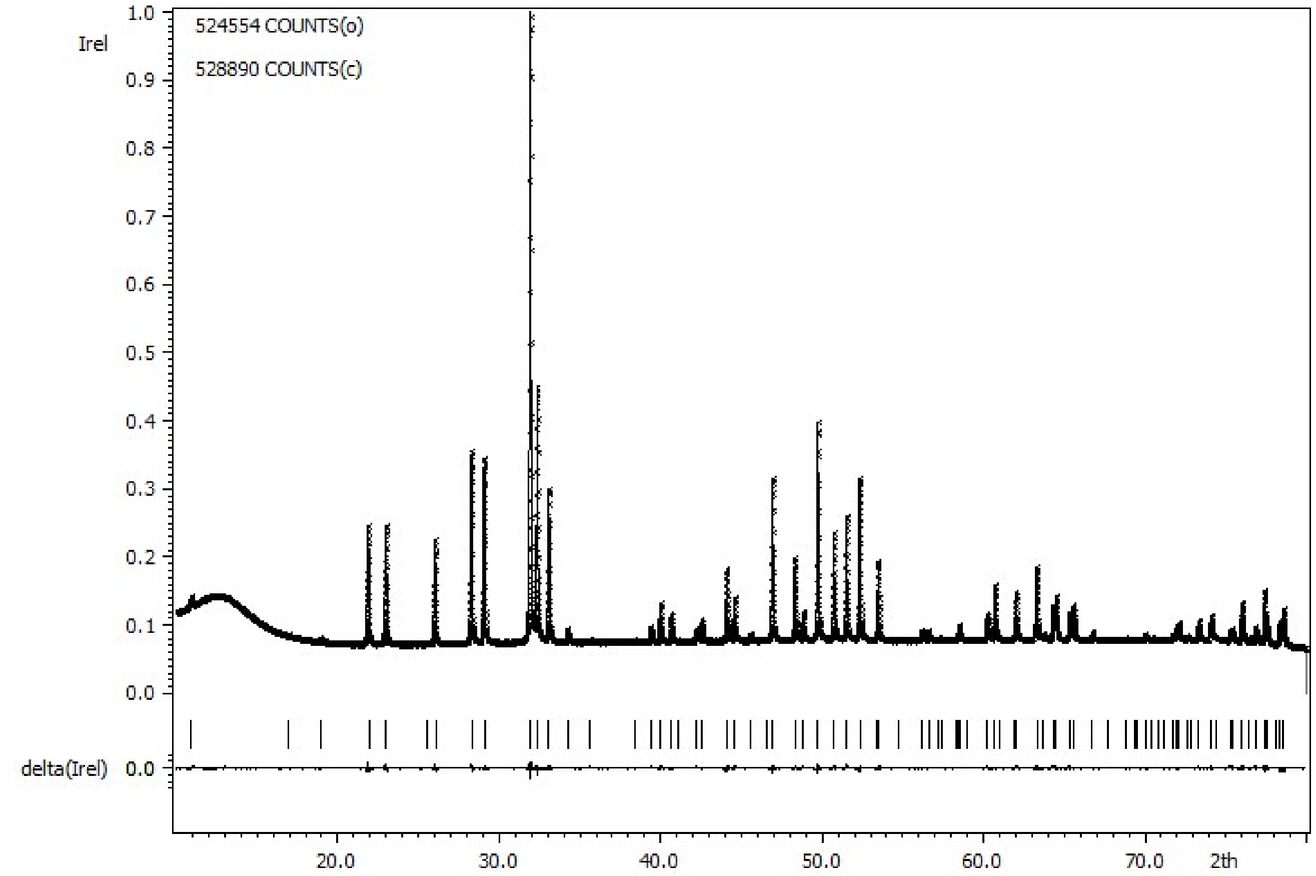

Figure S2. X-ray powder diffraction pattern of $Tb_8Ca_2(SiO_4)_6O_2$ (**2**). Observed (crosses), calculated (solid line) and difference (solid line below) plots. Positions of Bragg reflections are shown as strokes underneath.

Table S4. Crystal structure refinement data for **2**.

Temperature (K)	293 K
Wavelength (Å)	1.5406
Space group	$P6_3/m$
a (Å)	9.3918(1)
c (Å)	6.8552(1)
V (Å^3)	523.65(1)
Z	1
2θ range (deg.)	10 – 80
R_{wp}	0.009
R_{all}	0.013
ΔF_{max}, ΔF_{min} (e Å^{-3})	0.34, -0.23

Table S5. Atomic parameters and thermal displacement parameters (Å^2) in **2**.

Atom	Tb1, Ca1	Tb2, Ca2	Si	O1	O2	O3	O4
Site	4f	6h	6h	6h	6h	12i	2b
SOF	0.583(4), 0.417(4)	0.944(4), 0.056(4)	1	1	1	1	1
x	1/3	0.0064(2)	0.4001(4)	0.3211(8)	0.6032(9)	0.3385(5)	0
y	2/3	0.23920(13)	0.3725(5)	0.4922(8)	0.4755(8)	0.2499(5)	0
z	0.0009(5)	¼	1/4	1/4	1/4	0.0616(6)	0.25
U_{iso}	0.0156(8)	0.0102(10)	0.0097(14)	0.0106(17)	0.0106(17)	0.0106(17)	0.0106(17)

Table S6. Selected interatomic distances (Å) and angles (degrees) in **2**.

Tb1,Ca1-O1	2.330(6)	3x	Si-O1	1.627(11)	
Tb1,Ca1-O2	2.428(7)	3x	Si-O2	1.652(8)	
Tb1,Ca1-O3	2.808(4)	3x	Si-O3	1.631(5)	2x
Tb2,Ca2-O1	2.713(6)		O1-Si-O2	112.7(4)	
Tb2,Ca2-O2	2.375(12)		O1-Si-O3	111.3(3)	2x
Tb2,Ca2-O3	2.304(4)	2x	O2-Si-O3	108.2(3)	2x
Tb2,Ca2-O3	2.462(4)	2x	O3-Si-O3	104.7(3)	
Tb2,Ca2-O4	2.2169(16)				

Table S7. Crystal field parameters (in Wybourne notation) derived in the program CONCORD for Tb^{3+} using experimental atomic coordinates of the coordination polyhedrons of Tb1 and Tb2 sites in **2** (Table S5). Partial charges on the silicate oxygen atoms and on the intrachannel oxygen atom are -0.5 and -1.0 respectively.

Parameter	Value (cm^{-1})		
	Tb1 ($a \rightarrow x$, $c \rightarrow z$)	Tb2 ($a \rightarrow x$, $c \rightarrow z$)	Tb2 (z axis along easy magnetization axis)
B_{20}	767.59753	-454.31398	1046.486
B_{22}		-465.48025	56.27758
B_{40}	-167.38268	168.71902	217.17658
B_{42}		10.6668	-81.14088
B_{43}	-61.17901		
B_{44}		-134.91307	22.11683
B_{60}	-22.80747	-18.57463	40.92915
B_{62}		-24.41857	-12.8162
B_{63}	-10.89772		
B_{64}		6.55888	8.78223
B_{66}	2.48669	15.63302	9.03744
B_{21}			
B_{41}			
B_{61}			
B_{65}			
B_{21}'			93.71102
B_{22}'		-489.52972	
B_{41}'			186.83395
B_{42}'		166.27464	
B_{43}'	69.18642		-61.4633
B_{44}'		95.62813	
B_{61}'			12.53713
B_{62}'		-13.79711	
B_{63}'	21.53327		1.87805
B_{64}'		-8.83184	
B_{65}'			-7.23612
B_{66}'	8.42907	-7.01231	

Table S8. Modeling with the program PHI using crystal field parameters listed in Table S7. The energies of the ground multiplet $^7F_6$ are shown only.

Tb1

Energy (cm^{-1})	$\|M_J\|$ (%)
0	6 (99.97)
1.30025E-4	6 (99.85)
141.334	5 (99.36)
141.334	5 (99.36)
251.308	4 (99.09)
251.308	4 (99.09)
329.615	3 (98.32)
331.562	3 (99.65)
382.614	2 (98.82)
382.614	2 (98.82)
411.683	1 (98.70)
411.683	1 (98.70)
420.999	0 (98.66)

Tb2

Energy (cm^{-1})	$\|M_J\|$ (%)
0	6 (99.76)
2.56691E-5	6 (99.76)
131.399	5 (99.12)
131.399	5 (99.12)
258.227	4 (98.48)
258.282	4 (98.52)
377.011	3 (98.27)
377.461	3 (98.01)
477.873	2 (98.82)
478.508	2 (96.33)
538.13	1 (99.19)
561.09	1 (98.83)
576.718	0 (97.21)

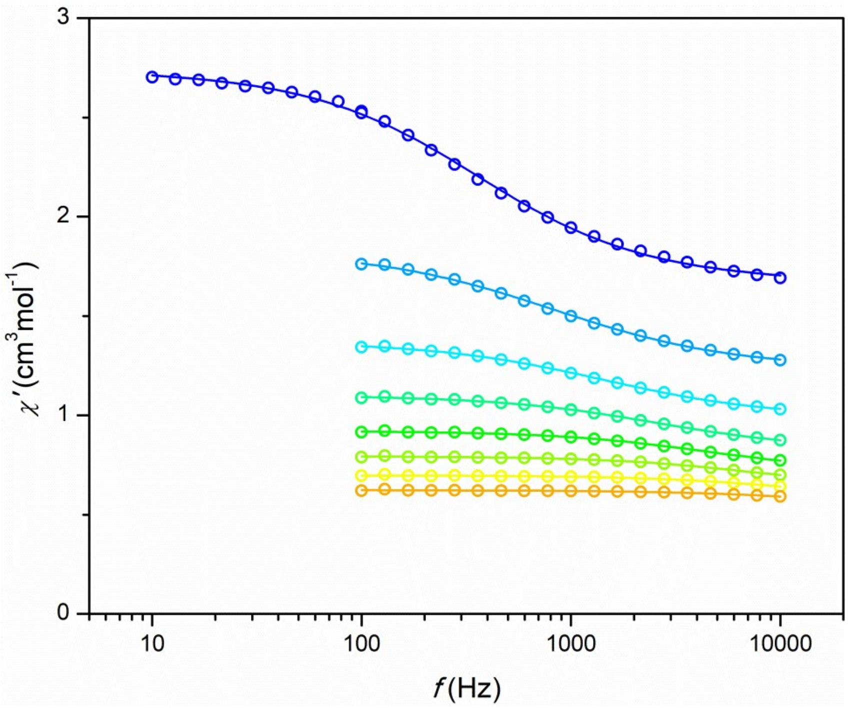

(a)

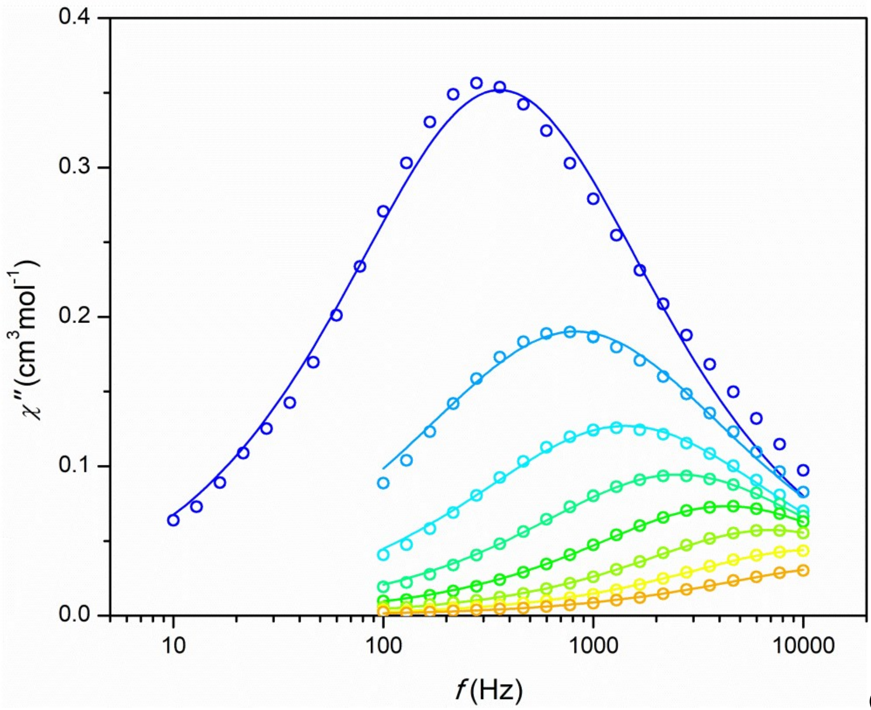

(b)

Figure S3. Frequency dependence of ac susceptibility per mol of Tb for **1** at different temperatures under a field of 1 kOe. (a) – in-phase susceptibility χ', (b) – out-of-phase susceptibility χ''. Symbols – experimental points, lines – fitting. Color designation: blue – green – red – orange, T = 2 – 16 K (step 2 K), respectively.

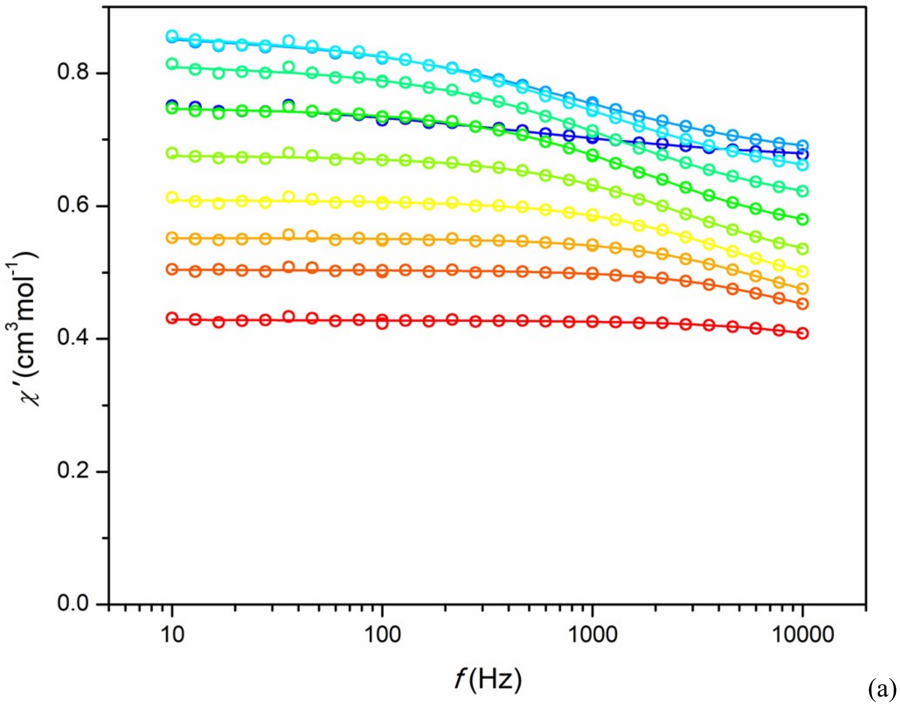

(a)

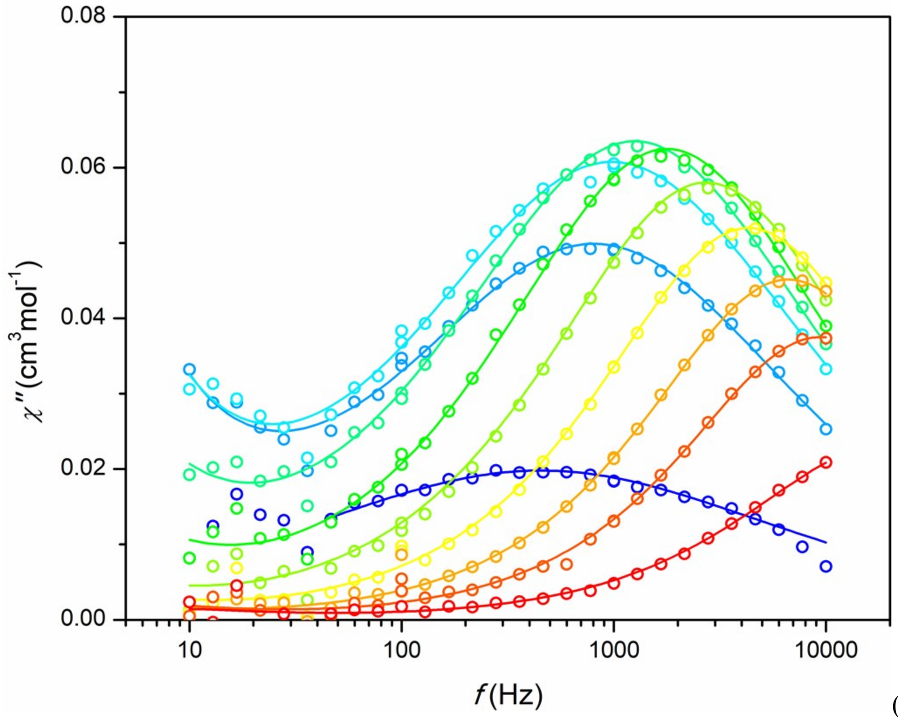

(b)

Figure S4. Frequency dependence of ac susceptibility per mol of Tb for **1** at different temperatures under a field of 8 kOe. (a) – in-phase susceptibility χ', (b) – out-of-phase susceptibility χ''. Symbols – experimental points, lines – fitting. Color designation: blue – green – yellow – red, T = 2 – 18 K (step 2 K) and T = 24 K, respectively.

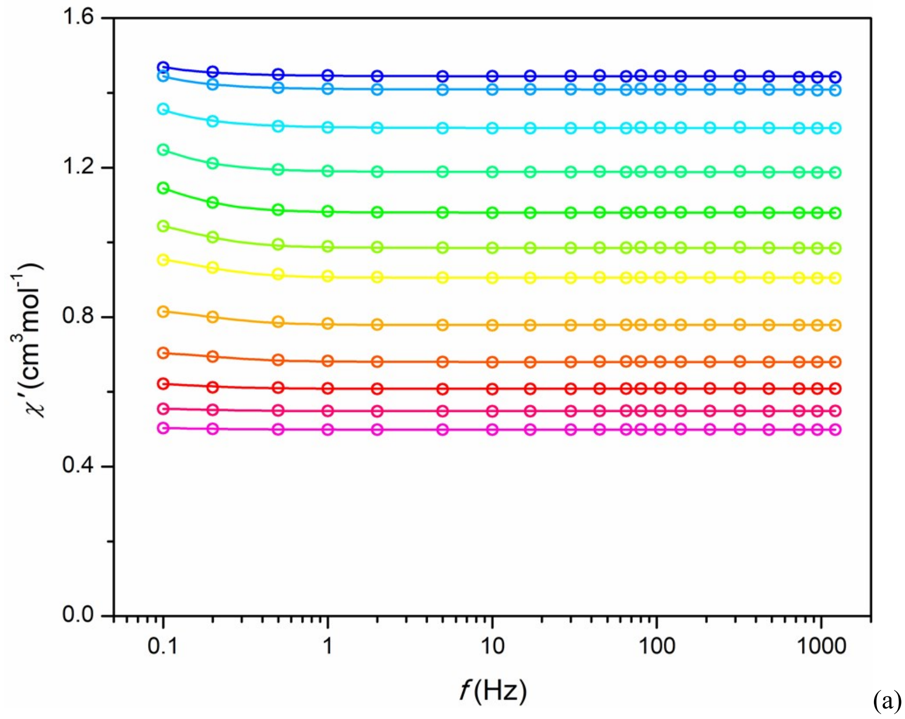

(a)

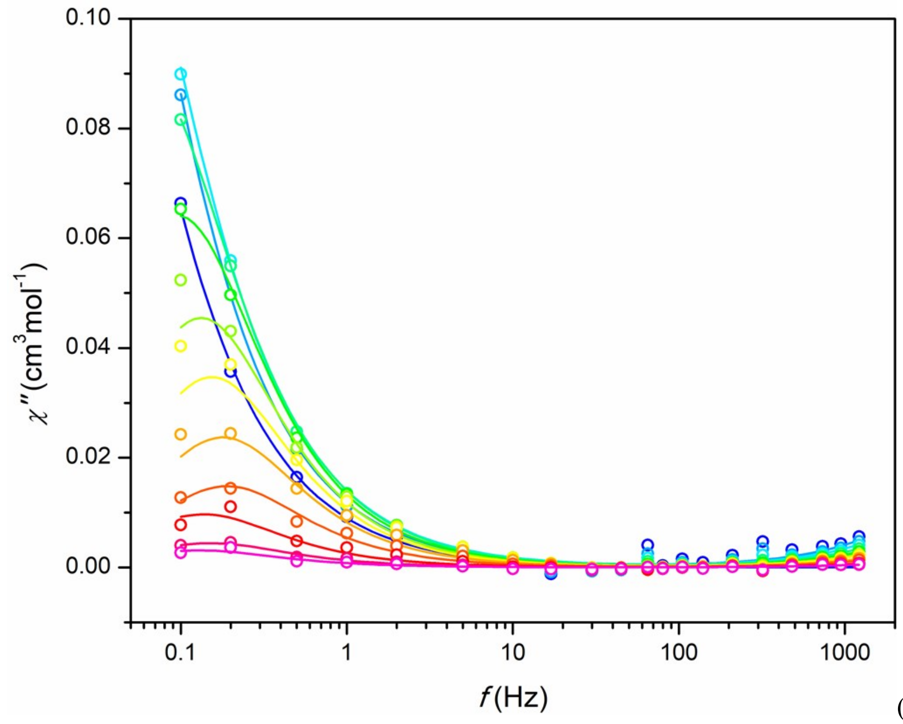

(b)

Figure S5. Frequency dependence of ac susceptibility per mol of Tb for **2** at different temperatures under a field of 4 kOe. (a) – in-phase susceptibility χ', (b) – out-of-phase susceptibility χ''. Symbols – experimental points, lines – fitting. Color designation: blue – green – orange – magenta, T = 2 – 8 K (step 1 K) and T = 10 – 18 K (Step 2 K), respectively.

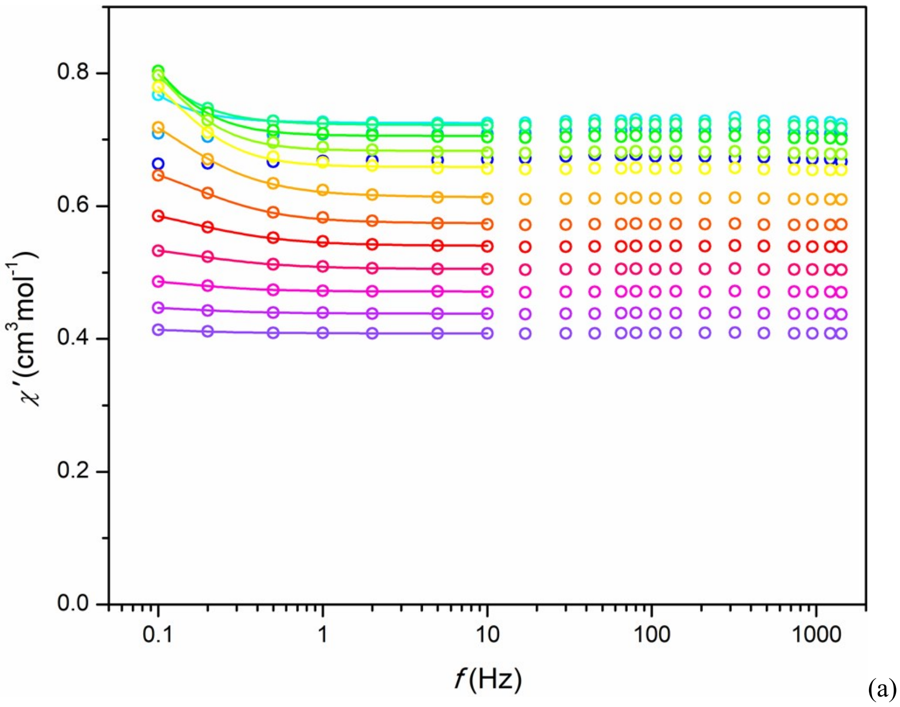

(a)

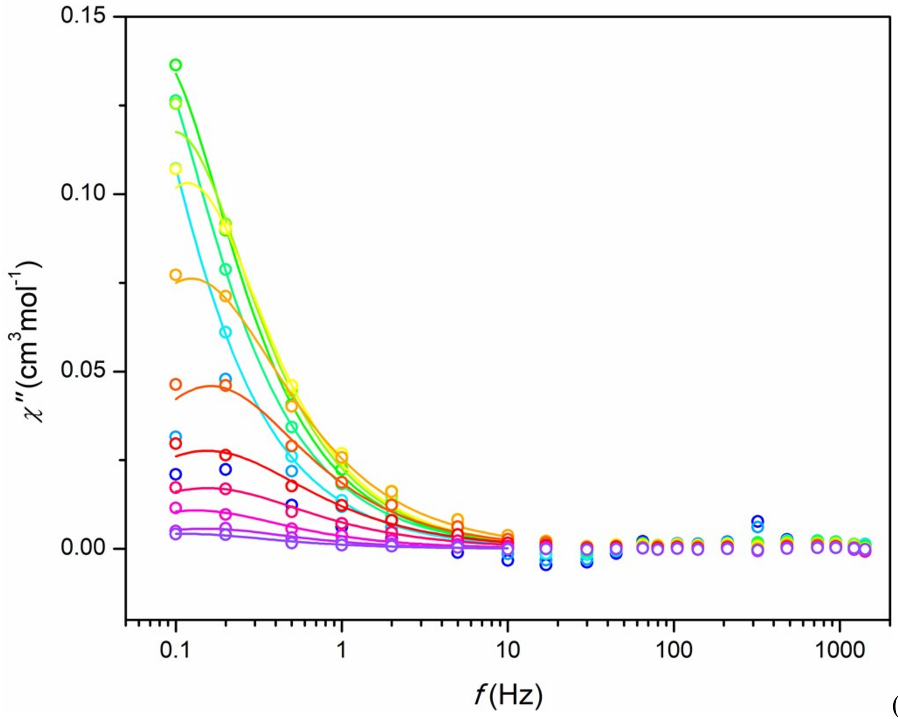

(b)

Figure S6. Frequency dependence of ac susceptibility per mol of Tb for **2** at different temperatures under a field of 8 kOe. (a) – in-phase susceptibility χ', (b) – out-of-phase susceptibility χ''. Symbols – experimental points, lines – fitting. Color designation: blue – green – orange – magenta – violet, T = 2 – 8 K (step 1 K) and T = 10 – 22 K (Step 2 K), respectively.
